# A Nomogram Based on Radiomics with Mammography Texture Analysis for the Prognostic Prediction in Patients with Triple-Negative Breast Cancer

**DOI:** 10.1155/2020/5418364

**Published:** 2020-08-25

**Authors:** Xian Jiang, Xiuhe Zou, Jing Sun, Aiping Zheng, Chao Su

**Affiliations:** ^1^Clinical Research Center for Breast, State Key Laboratory of Biotherapy, West China Hospital, Sichuan University, Chengdu, China; ^2^Laboratory of Tumor Targeted and Immune Therapy, Clinical Research Center for Breast, State Key Laboratory of Biotherapy, West China Hospital, Sichuan University and Collaborative Innovation Center, Chengdu, China; ^3^Department of Thyroid Surgery, West China Hospital, Sichuan University, Chengdu, China; ^4^Department of Integrated Chinese and Western Medicine, Qingdao Central Hospital, Qingdao University, Qingdao, Shandong, China; ^5^West China School of Medicine, Sichuan University, Chengdu, China; ^6^State Key Laboratory of Biotherapy and Cancer Center/Collaborative Innovation Center for Biotherapy, West China Hospital, Sichuan University, Chengdu, China

## Abstract

**Objectives:**

To develop and validate a radiomics-based nomogram with texture features from mammography for the prognostic prediction in patients with early-stage triple-negative breast cancer (TNBC).

**Methods:**

The study included 200 consecutive patients with TNBC (training cohort: *n* = 133, validation cohort: *n* = 67). A total of 136 mammography-derived textural features were extracted, and LASSO (least absolute shrinkage and selection operator) was applied to select features for building the radiomics score (Rad-score). After univariate and multivariate logistic regression, a radiomics-based nomogram was constructed with independent prognostic factors. The discrimination and calibration power were assessed, and further the clinical applicability of the nomograms was evaluated.

**Results:**

Among the 136 mammography-derived textural features, fourteen were used to build the Rad-score after LASSO regression. A radiomics nomogram that incorporates Rad-score and pN stage was constructed. This nomogram achieved a C-index of 0.873 (95% CI: 0.758–0.989) for predicting iDFS (invasive disease-free survival), which outperformed the clinical model. Moreover, it is feasible to stratify patients into high-risk and low-risk groups based on the optimal cut-off point of Rad-score. The validations of the nomogram confirmed favorable discrimination and considerable predictive efficiency.

**Conclusions:**

The radiomics nomogram that incorporates Rad-score and pN stage exhibited favorable performance in the prediction of iDFS in patients with early-stage TNBCs.

## 1. Introduction

Breast cancer, the most frequently diagnosed malignancy, is the leading cause of cancer-related deaths among women [1]. Triple-negative breast cancer (TNBC), accounting for 15–20% of breast cancers [2], does not benefit from endocrine therapy or classic targeted therapy due to the absence of estrogen receptor (ER), progesterone receptor (PR), and the human epidermal growth factor receptor (HER-2) gene amplification. Despite the attempts of novel therapeutic agents such as immune checkpoint inhibitors [3] and poly (ADP-ribose) polymerase inhibitors [4], traditional cytotoxic chemotherapy is still the mainstream systemic treatment option for TNBC [5], while the clinical outcomes remain the poorest among all molecular subtypes of breast cancers.

Nomogram is a feasible and efficacious statistical predictive tool that incorporates multiple variables of value [6, 7]. Seeking new prognostic factors and embedding them into nomograms is an important research method in the prediction of clinical outcomes. To date, nomogram predictions for the survival of TNBCs merely considered conventional clinical and pathological risk factors [8–10]. While efforts have been made in adding novel prognostic factors such as the expression of HIF-1*α* and c-myc to nomograms [11], imaging features have not yet been analyzed. Nowadays, the advent of deep-learning-based “radiomics” technology has allowed for the high-throughput extraction of quantitative imaging features from images, thus enhancing the accuracy of diagnosis and prognostic prediction, especially for malignancies [12]. Radiomics-based methods are being widely applied in discrimination of confusing lesions on the images [13, 14]. Mammography, ultrasound, and MRI are the most important diagnostic imaging modalities in the management of breast cancers. And imaging-based radiomics nomograms have been constructed in the prediction of axillary lymph node metastasis in early-stage breast cancer [12, 15, 16].

Mammography, mainly displayed in craniocaudal (CC) and mediolateral oblique (MLO) views, has long been a routine screening method for early detection of breast cancers, typically through detection of characteristic masses, microcalcifications, and/or architectural distortions. Apart from distinct biological properties and clinical activation, TNBCs might be distinguished from non-TNBCs with radiomics features based on mammography [17]. More importantly, mammographic features can further help in differentiating basal-like and normal-like subtypes of TNBCs [18]. These facts suggested that mammographic radiomics features might be a potential prognostic factor for TNBCs.

So far, there has been no radiomics-based study for the prognostic prediction of TNBC to the best of our knowledge. Therefore, this study analyzes the prognostic value of mammography textures using deep-learning strategies and constructs an optimized nomogram for the prognostic prediction of TNBCs.

## 2. Methods

This study was approved by the West China Hospital Research Ethics Committee (No. 2019[887]). The study only involved retrospective analysis of anonymous data, and in consequence the requirement for informed consent was waived.

### 2.1. Patient Selection

Between April 14, 2010, and April 17, 2017, a total of 200 consecutive patients with TNBC who were treated at West China Hospital of Sichuan University were retrospectively identified from hospital database. The inclusion criteria were pathologically diagnosed TNBCs with mammography performed within 3 months before surgery. In the determination of TNBCs, statuses of ER, PR, and HER-2 were tested with immunostaining. Uncertain status of HER-2 amplification was confirmed by fluorescence in situ hybridization. The exclusion criteria were as follows: a. ductal carcinoma in situ (DCIS) or Paget's disease without invasive elements; *b*. excisional biopsy prior to mammography; *c*. neoadjuvant therapy prior to mammography; *d*. nonmass lesions, i.e., abnormities visible on either mammographic views, which could not be characterized as a distinct mass because of lack of a conspicuous margin or shape [19], including (1) calcification without clear boundary, (2) architectural distortion, and (3) focal asymmetric density; *e*. patients with negative mammography; *f*. recurrent or metastatic diseases.

The included patients were randomly divided into the training (*n* = 133) and validation (*n* = 67) datasets at a ratio of 2 : 1 using random number table (Supplementary Table 1). The clinicopathological features and treatment strategies of the patients were retrieved from medical records, including age, clinical stage, WHO classification, pathological type, Ki-67 expression, type of surgery, radiation therapy, and chemotherapy.

### 2.2. Follow-Up

The primary end point of this study was invasive disease-free survival (iDFS). After surgeries, patients were routinely followed up every six months for the first five years and annually ever after. IDFS was defined as the period from the date of diagnosis until the date of ipsilateral invasive breast cancer recurrence, ipsilateral locoregional invasive breast cancer recurrence, contralateral invasive breast cancer, distant recurrence/metastasis, death from any cause, and/or the date of last follow-up.

### 2.3. Image Acquisition and Texture Feature Extraction

Mammography images were acquired through Mammomat Novation DR systems (SIEMENS, German). Meanwhile, craniocaudal (CC) and mediolateral oblique (MLO) projections of both breasts were acquired for each patient.

**Figure 1:**
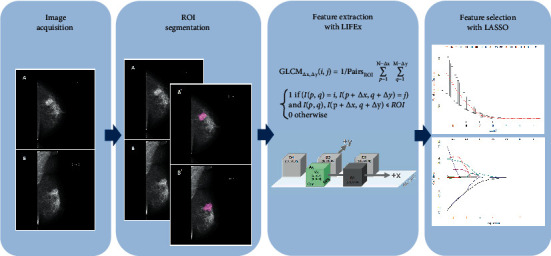
Workflow of radiomic signature building. Abbreviation: ROI = region of interest; LIFEx = Local Image Feature Extraction; LASSO = the least absolute shrinkage and selection operator.

Two participants (XJ and JY) who were blinded to patient information independently extracted texture features of mammography images using Local Image Feature Extraction (LIFEx) software (http://www.lifexsoft.org, version 5.10) and were supervised by a senior breast radiologist in case of controversies [20]. Following the software instructions (supplementary data), the regions of interests (ROIs) on both CC and MLO views were carefully drawn along the edge of lesions (Figure 1). Sixty-eight features of each mammographic view were automatically extracted, and 136 features of both CC and MLO projections were used to form the radiomic statistical dataset for subsequent machine-learning analysis. The extracted texture features by LIFEx include the grey level co-occurrence matrix (GLCM), the neighborhood grey-level different matrix (NGLDM), the grey-level run length matrix (GLRLM), and the grey-level zone length matrix (GLZLM). Original data of texture features extracted from 200 patients' mammography images is provided in the Supplementary Table 2. Detailed description of features is provided in the supplementary data.

### 2.4. Selection of Radiomics Signatures

A total of 136 textural features were extracted for each patient. The logistic regression model with least absolute shrinkage and selection operator (LASSO) was adopted to select value features for clinical outcomes with nonzero coefficients (Figure 1) [21]. The calculation formula of Rad-score was subsequently constructed with a linear combination of the selected features of value that were weighted by their respective coefficients [22, 23].

### 2.5. Construction, Assessment, and Validation of the Radiomics Nomogram

Univariate and multivariate analyses were conducted with Cox proportional hazards regression model. Firstly, a univariate analysis of Rad-score and clinicopathological features for iDFS prediction was performed within the training dataset. For each parameter, hazard ratios (HRs) and 95% confidence intervals (95% CIs) were calculated, and significant variables (*p* < 0.05) in univariate analysis were then tested in backward stepwise selection in the multivariate logistic regression model. Upon the basis of the multivariate regression model, the independent predictive factors (*p* < 0.05) of iDFS were incorporated in the ultimate nomogram, through which a risk score was calculated for each patient.

In order to assess the predictive efficacy of nomogram, the calibration curve was drawn to evaluate the calibration of nomogram and the concordance index (C-index) was applied to further assess its performance.

The internal validation of the nomogram was performed with the validation dataset. Each patient in the validation cohort received a Rad-score with the established formula. The calibration curve and C-index calculation were performed subsequently. Moreover, Kaplan–Meier (K–M) survival curve analysis of iDFS based on the median value of the radiomics nomogram was performed to stratify patients into high- and low-risk subgroups.

### 2.6. Clinical Utility Evaluation of Radiomics Nomogram

Decision curve analysis (DCA) was conducted to evaluate the clinical significance of radiomics nomogram in predicting iDFS in TNBC patients. More specifically, the net benefits at ranges of threshold probabilities were calculated in the combined training and validation cohorts.

### 2.7. Statistical Analysis

The comparisons of clinicopathological features between training and validation cohorts were assessed by Student's *t*-test or Mann–Whitney *U* test for continuous variables and Pearson's chi-squared test or Fisher's exact test for categorical variables. The survival curves were displayed with Kaplan–Meier method and differences in survival were examined using the log-rank test. All statistical analysis were performed using *R* software (version 3.5.2). The *R* packages implemented included glmnet, psych, rms, Hmisc, survival, survminer, grid, Lattice, Formula, ggplot2, nomogramEx, tidyverse, dplyr, tidyr, rmda, devtools, rmda, and MASS. A two-tailed *p* < 0.05 was considered to be statistically significant.

## 3. Result

### 3.1. Patient Characteristics and iDFS

A total of 200 TNBC patients were included for data analysis and patient characteristics in training and validation cohorts are summarized in Table 1. There were no statistically significant differences in the follow-up duration, clinical-pathological characteristics, or treatment strategies between the two cohorts.

As of the last follow-up, 17 patients (8.50%) had experienced disease relapse or death. The mean iDFS was 17.58 months and the median iDFS was 16.23 (3.10 to 36.43) months. The 1-, 2-, 5-, and 8-year cumulative iDFS of all patients were 2.50% (5/200), 7.00% (14/200), 8.50% (17/200), and 8.50% (17/200), respectively.

### 3.2. Construction of Radiomics Score

**Figure 2:**
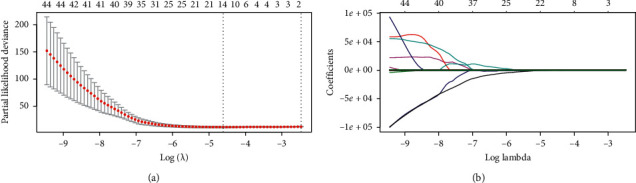
LASSO selection and the predictive efficacy of radiomics features. (a). Tuning parameter (*λ*) selection with minimum criteria-based 10-fold cross-validation in the LASSO model. Binomial deviances (*y*-axis) were plotted as a function of log (*λ*) (lower *x*-axis), and the upper *x*-axis represents the average number of predictors. The dotted vertical lines were drawn at the optimal values of *λ* and the value that gave the minimum average binomial deviance was used to select radiomics features. The optimal *λ* value of 0.01 (log (*λ*) = −4.610) was selected. (b) LASSO coefficient profiles of the 136 texture features. Each colored curve represents the trajectory of the change of an independent variable. At the value selected using 10-fold cross-validation, the optimal *λ* resulted in fourteen coefficients.

Among the 136 mammography-derived textural features, fourteen were used to build the Rad-score after LASSO regression (Figure 2). The equation of Rad-score was as follows:

(1) $$\begin{array}{c} \text{Rad}-\text{score}=\text{CONVENTIONAL}\_\#\text{std}\_C^{*}\left(-0.00132048\right)\\ +\text{SHAPE}\_\text{Volume }\left(mL\right)\_CC^{*}\left(0.004541706\right)\\ +\text{SHAPE\_Volume }\left(\#vx\right)\_CC^{*}\left(2.016478E-19\right)\\ +\text{GLRLM}\_\text{SRE}\_CC^{*}\left(-74.75245\right)\\ +\text{NGLDM}\_\text{Contrast}\_CC^{*}\left(-5.214166\right)\\ +\text{NGLDM}\_\text{Busyness}\_CC^{*}\left(7.177503\right)\\ +\text{CONVENTIONAL}\_\#\min\;\_MLO^{*}\left(0.0006480479\right)\\ +\text{HISTO}\_\text{Skewness}\_MLO^{*}\left(-0.4479433\right)\\ +\text{GLCM}\_\text{Correlation}\_MLO^{*}\left(-0.7861222\right)\\ +\text{GLCM}\_\text{Entropy}\_\log10\_MLO^{*}\left(0.4937121\right)\\ +\text{GLCM}\_\text{Entropy}\_\log2\;\left(=\text{Joint entropy}\right)\_MLO^{*}\left(0.0000005908434\right)\\ +\text{GLRLM}\_\text{GLNU}\_MLO^{*}\left(-0.0004946444\right)\\ +\text{GLZLM}\_\text{SZE}\_MLO^{*}\left(20.57717\right)\\ +\text{GLZLM}\_\text{SZHGE}\_MLO^{*}\left(0.00003251064\right). \end{array}$$

### 3.3. Development and Validation of Nomogram

**Figure 3:**
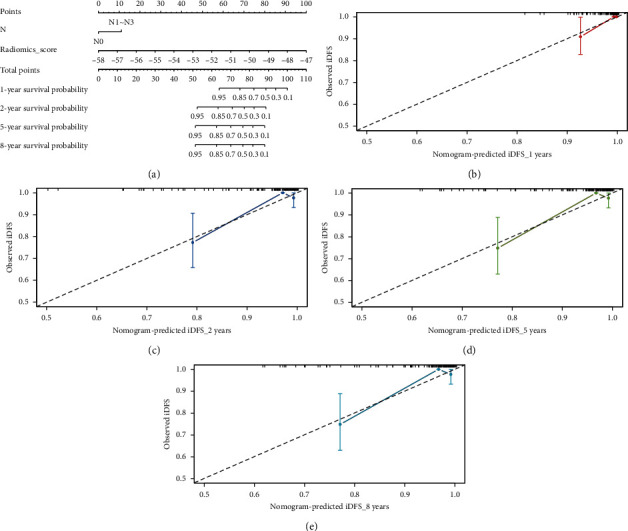
Radiomics nomogram to estimate iDFS for patients with triple-negative breast cancers and its discrimination performance. (a) The radiomics nomogram was developed by incorporating pN stage and radiomics score. b−e. Calibration curves of the nomogram for the estimation of 1-year (b), 2-year (c), 5-year (d), and 8-year (e) iDFS in the training cohort. The diagonal line represents a perfect match between the predicted (*x*-axis) and actual (*y*-axis) probabilities, and the colored line represents the predictive performance of the nomogram. The closeness between the two lines indicates the predictive accuracy of the nomogram.

Univariate Cox regression model analysis indicated that pN stage (HR: 3.964, 95% CI: 1.258–12.490, *p*=0.019) and Rad-score (HR: 3.071, 95% CI: 1.949–4.840, *p* < 0.001) were associated with iDFS in TNBC patients (Table 2). Subsequently, multivariate Cox regression analysis confirmed pN stage (HR: 3.898, 95% CI: 1.181–12.867, *p*=0.026) and Rad-score (HR: 3.052, 95% CI: 1.868–4.985, *p* < 0.001) as independent risk factors for iDFS. Accordingly, the nomogram was constructed to quantify probability 1-, 2-, 5-, and 8-year survival (Figure 3(a)). As assessed by the calibration curves, the nomogram revealed good calibration in the prediction of iDFS (Figures 3(b)–3(e)). The C-index for the radiomics nomogram was 0.873 (95% CI: 0.758–0.989) in the training cohort and 0.944 (95% CI: 0.883–1.004) in the validation cohort, while for the clinical nomogram (*N* stage), it was 0.668 (95% CI: 0.531–0.805) in the training and 0.761 (95% CI: 0.608–0.913) in the validation cohort, indicating a better efficacy of radiomics nomogram than clinical nomogram in predicting iDFS.

**Figure 4:**
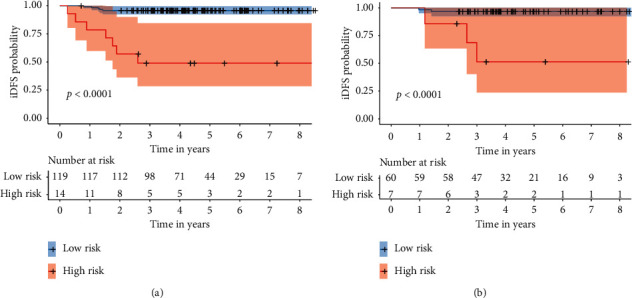
Kaplan–Meier survival analyses of high-risk and low-risk patients in the training (a) and validation cohort (b).

We were able to stratify patients into high-risk and low-risk groups based on the optimal cut-off point obtained by the “surv_cutpoint” function of the “survminer” *R* package [24, 25]. Patients with a total score higher than or equal to -51.53 were identified as high-risk patients (*n* = 21), and those with a total score less than -51.53 were classified as low-risk patients (*n* = 179). The verification with K-M survival curves showed that the iDFS in the high-risk group was much lower than that in the low-risk group in both the training (*p* < 0.0001) and validation (*p* < 0.0001) cohorts (Figure 4).

### 3.4. Clinical Utility

**Figure 5:**
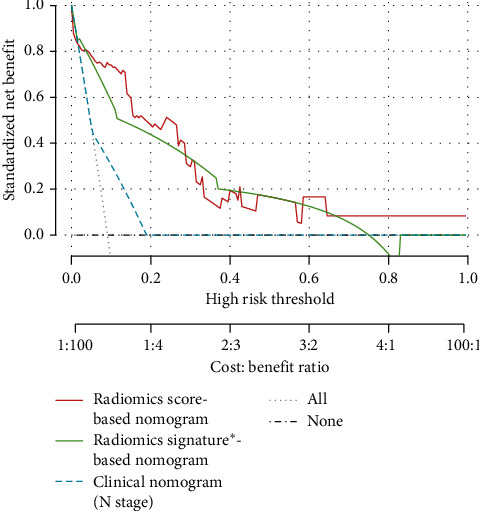
Decision curve analysis for each model in survival prediction in patients with triple-negative breast cancers (TNBCs). The *x*-axis represents the threshold probability and the *y*-axis represents the net benefit. The grey line represents the assumption that all patients had experienced events of invasive disease. The black horizontal line represents the assumption that no patient had invasive disease. The decision curves show that using the radiomics nomograms to predict survival adds more benefit for TNBC patients than all other models. ^*∗*^Radiomics score binarily classified as high- and low-risk groups.

The DCAs for the radiomics nomogram and clinical nomogram are presented in Figure 5. The radiomics nomogram adds more net benefit than the “treat all” or “treat none” strategies without limitation on the threshold probability.

## 4. Discussion

Due to an advanced histological grade, a more aggressive behavior, and the lack of effective therapeutic targets, the clinical outcomes of TNBCs remain the poorest among all molecular subtypes of breast cancers [26]. The recurrence pattern of TNBCs is distinct from non-TNBCs. The risk of disease relapse and death steadily continues for seventeen years after diagnosis in non-TNBCs [27]. However, in patients with TNBCs, the risk of recurrence reached its peak in the first three years after diagnosis and declines thereafter. Recurrence is unlikely to occur in patients who remain disease-free over eight years after diagnosis [27]. Moreover, TNBCs are highly heterogeneous and they respond variously to standard chemo-regimens, resulting in a comparatively wide range of survival [28]. Therefore, a model that validly predicts the survival of TNBCs has significant clinical value. In this study, we assessed the value of radiomics features of mammography in the prognostic prediction of TNBCs, and the results revealed a desired effect. Accordingly, we further established and validated a radiomics-based nomogram to accurately predict the iDFS in TNBC patients. The nomogram contains two indicators, namely, Rad-score and pN stage. With the addition of mammography-derived radiomic score, the nomogram significantly improved the predictive efficiency compared to the existing predictive models.

Mammography has been adopted as a screening modality since 1960s and is currently accepted as the most effective screening approach for breast cancer [29, 30]. There are several discriminative mammographic findings on TNBCs from non-TNBCs. The majorities of TNBCs appear as a mass on mammograms [31]. In a retrospective study with 198 premenopausal patients with breast cancer, all TNBCs were associated with a mass (*n* = 33) while 55% of HER-2+ cancers and 48% of ER + cancers were related [32]. Moreover, TNBCs tend to be less frequently associated with calcifications on mammography compared to non-TNBCs [32], potentially because TNBCs progress rapidly to invasive disease without a period of precancerous disease with in situ components that is sufficient to allow calcifications to form [31]. In a study involving 91 TNBC patients, analyses of mammographic features suggested that mass margins were significantly different between basal-like TNBCs and normal-like TNBCs. More specifically, margins of basal-like TNBCs were microlobulated or speculated, whereas those normal-like TNBCs were more likely to be microlobulated [18]. These results suggest that the mammographic presentation of tumors may reflect the histological characteristics and biological behavior of the tumors with sophisticated mechanism.

Despite the wide popularity and the huge number of examinations, which makes mammography a potential data reservoir in big data medicine, the in-depth information hidden in the images has not been taken advantage of. Nowadays, the advent of deep learning-based radiomics technology has improved the accuracy of disease diagnosis and prognostic prediction [12]. Radiomics is the process of high-throughput extraction of a large number of image features, which converts traditional medical images into high-dimensional data that can be mined, and allows the subsequent quantitative analysis of these data [33]. It helps in the identification of tumor types, noninvasively and quantitatively evaluates tumor biological heterogeneity [34], and therefore optimizes disease detection, diagnosis, treatment response prediction, and prognosis evaluation to promote clinical decision-making. At present, radiomics is mainly applied into the management of malignant tumors, such as liver cancer [35, 36], lung cancer [37], and glioblastoma [38].

In breast cancers, MRI-based radiomics has been evaluated in the prediction of response to neoadjuvant therapy [39–41], prediction of sentinel lymph node metastasis [42], and recognition of molecular subtypes [43]. Moreover, ultrasound-based radiomics has been applied in the prediction of axillary lymph node metastasis [12] and the differential diagnosis between TNBC and fibroadenoma [44]. Mammography-based radiomics has been adopted in breast cancer diagnosis [45]. However, few studies have applied radiomics in prognostic prediction of patients with breast cancer. This study analyzed the prognostic value of mammography textures and constructed an optimized nomogram that incorporated Rad-score and clinical features (pN stage) for the prognostic prediction of TNBCs. According to the results of univariate and multivariate regression, Rad-score rather than *T* stage remained an independent prognostic factor apart from pN stage. The Rad-score was constructed with parameters of the grey level co-occurrence matrix (GLCM), the neighborhood grey-level different matrix (NGLDM), the grey-level run length matrix (GLRLM), and the grey-level zone length matrix (GLZLM). These parameters take into account number, distance, angle, etc. (supplementary data). The Rad-score and the according nomogram exhibited favorable discrimination and considerable predictive efficiency. Based on the Rad-score and the according nomogram, we can predict the prognosis of patients according to the texture features of tumor combined with pN stage and optimize the surgical mode and chemotherapy accordingly. Rad-score successfully identified high-risk patients with poor survival outcomes who need more intensive treatment. Compared to long-term outcome overall survival, iDFS is an end point that avoids extended follow-up and enables early adjustment of treatment. Therefore, our study may provide a more effective tool for making early personalized treatment.

There are several limitations of our study. First, the retrospective nature of the study design might inevitably bias the patient selection. Second, this is a single-center study and the sample size was relatively small. Although the incidence of breast cancers remains the highest among malignancies of all sites in women, the mortality is comparatively lower. In 2019, breast cancer accounts for 30% of the estimated new cases of cancers in the United States, while the estimated deaths only account for 15% [46]. As a consequence in our situation, the number of iDFS events was even smaller which may weaken the statistical power of the current model. Thus, the nomogram should be further validated in prospective studies with larger sample size and longer follow-up. Third, we only included TNBC patients with a definite mass on mammogram to allow a ROI to be drawn. Therefore, our nomogram is not applied to TNBCs with negative mammograms and those with lesions appeared as distortions or calcifications with no clear boundaries.

In conclusion, we established a novel nomogram that can effectively predict the iDFS in TNBC patients by incorporating mammography-based radiomics features into clinicopathological variables. This nomogram mainly benefits primary TNBCs with a mass-like lesion on mammography. Still, future work is required to evaluate its reliability as a routine clinical tool.

## Figures and Tables

**Table 1:** Clinical and pathological characteristics of patients in training and validation cohorts.

	Training cohort (*n* = 133)	Validation cohort (*n* = 67)	*p* value
Age (years, mean ± SD)	49.22 ± 9.87	47.79 ± 10.10	0.196

BMI (kg/m [2], mean ± SD)	23.59 ± 3.32	22.88 ± 2.63	0.232

Follow-up (months, mean ± SD)	54.98 ± 21.72	54.39 ± 22.63	0.701

Menopausal status			0.889
Premenopausal	72	39
Postmenopausal	60	29

T Stage			0.651
T1	38	23
T2-4	92	42
Tx	3	2

N stage			0.452
N0	95	44
N1-3	37	23

WHO classification			0.276
1	0	0
2	6	6
3	108	47
NA	19	14

Ki-67 status			0.710
<14%	7	2
≥14%	126	65

Type of breast surgery			0.928
Mastectomy	120	60
Lumpectomy	6	4
NA	7	3

Neoadjuvant chemotherapy			1.000
Yes	13	7
No	120	60

Adjuvant chemotherapy			1.000
Yes	127	64
No	6	3

Adjuvant radiotherapy			0.468
Yes	30	19
No	103	48

SD: standard deviation; BMI: body mass index; NA: not available.

**Table 2:** Univariate and multivariate analysis of clinicopathological-radiomics characteristics for prognostic prediction in training cohort.

Variables	Univariate regression		Multivariate regression	
	HR (95% CI)	*p* value	HR (95% CI)	*p* value
Age (years)	1.007 (0.951–1.066)	0.812		
BMI (kg/m^2^)	0.950 (0.793–1.138)	0.575		
Menopausal status	0.373 (0.101–1.377)	0.139		
pT stage	2.199 (0.482–10.040)	0.309		
pN stage	3.964 (1.258–12.490)	0.019^*∗*^	3.898 (1.181–12.867)	0.026^*∗*^
Histological type	3.886 × 10^−8^ (0-Inf)	0.998		
WHO classification	0.506 (0.065–3.956)	0.516		
Ki-67 status	2.643 × 10^7^ (0-inf)	0.998		
Type of breast surgery	1.750 (0.226–13.560)	0.592		
Neoadjuvant chemotherapy	1.774 (0.389–8.096)	0.459		
Adjuvant chemotherapy	0.534 (0.069–4.138)	0.548		
Adjuvant radiotherapy	1.117 (0.302–4.127)	0.868		
Radiomics score	3.071 (1.949–4.840)	1.331 × 10^−6^^*∗∗∗*^	3.052 (1.868–4.985)	8.380 × 10^−6^^*∗∗∗*^

^*∗*^ *p* < 0.05; ^*∗∗*^*p* < 0.001. HR: hazard ratio; BMI: body mass index.

## Data Availability

The raw data supporting the conclusions of this article will be made available by the authors, without undue reservation.

## Supplementary Materials

Supplementary Materials Supplementary data: image feature extraction process, description of 14 mammography-derived textural features. Supplementary Table 1: random number table. Supplementary Table 2: original data of texture features extracted from 200 patients' mammography images.
