# Supplementary material for: A Nomogram Based on Radiomics with Mammography Texture Analysis for the Prognostic Prediction in Patients with Triple-Negative Breast Cancer
Source: Contrast Media Mol Imaging. 2020 Aug 25;2020:5418364. doi: 10.1155/2020/5418364 (PMC7468630; doi:10.1155/2020/5418364)
Supplement: Supplementary Materials — Supplementary data: image feature extraction process, description of 14 mammography-derived textural features. Supplementary Table 1: random number table. Supplementary Table 2: original data of texture features extracted from 200 patients' mammography images. [file 5418364.f1.zip › 5418364.f1/supplementary data.pdf]

Supplementary data

Image feature extraction process:

1. Reading of the DICOM images for which index should be calculated;
2. Drawing the ROI:

To create a new ROI, click on "New" icon and select the 3D pencil. To draw a ROI, the instructions are given in the lower left-hand corner of the software, and we use the "Shift + left click" to draw;

3. Definition of the input parameters: default values can be changed, and parameters without default values should be set;
4. Definition of the output parameters of interest: index to be calculated should be selected;
5. Running the calculation: click RUN in the textural index setting window;
6. The folder containing the csv file listing the results is open.

Description of 14 mammography-derived textural features:

The grey level co-occurrence matrix (GLCM) takes into account the arrangements of pairs of voxels to calculate textural indices. The GLCM is calculated from 13 different directions in 3D with a δ-voxel distance relationship between neighbored voxels. The index value is the average of the index over the 13 directions in space (X, Y, Z). Seven textural indices are computed from this matrix.

GLCM_Correlation is the linear dependency of grey-levels in GLCM.

GLCM_Entropy_log10 is the randomness of grey-level voxel pairs.

GLCM_Entropy_log2 is the randomness of grey-level voxel pairs.

The neighborhood grey-level different matrix (NGLDM) corresponds to the difference of grey-levels between one voxel and its 26 neighbours in 3 dimensions (8 in 2D). Three texture indices can be computed from this matrix.

NGLDM_Contrast is the intensity difference between neighbouring regions.

NGLDM_Busyness is the spatial frequency of changes in intensity.

The grey-level run length matrix (GLRLM) gives the size of homogeneous runs for each grey level. This matrix is computed for the 13 different directions in 3D (4 in 2D) and for each of the 11 texture indices derived from this matrix, the 3D value is the average over the 13 directions in 3D (4 in 2D).

GLRLM_SRE (Short-Run Emphasis) is the distribution of the short homogeneous runs in an image.

GLRLM_GLNU (Gray-Level Non-Uniformity for run) is the nonuniformity of the grey-levels.

The grey-level zone length matrix (GLZLM) provides information on the size of homogeneous zones for each grey-level in 3 dimensions (or 2D). It is also named Grey Level Size Zone Matrix (GLSZM). From this matrix, 11 texture indices are computed.

GLZLM_SZE (Short-Zone Emphasis) is the distribution of the short homogeneous zones in an image.

GLZLM_SZHGE (Short-Zone High Gray-level Emphasis) is the distribution of the short

homogeneous zones with high grey-levels.

SHAPE_Volume (mL and voxels) is the Volume of Interest in mL and in voxels.
CONVENTIONALmin reflects the minimum of value (in choosen unit) in the Volume of Interest.
CONVENTIONAL_std is the standard deviation value of voxel values from the envelopes.
HISTO_Skewness is the asymmetry of the grey-level distribution in the histogram.

Supplementary table 1
Random number table

Supplementary table 2
Original data of texture features extracted from 200 patients’ mammography images
